# Performance Evaluation of Nonacosan-10-ol-Based Polyethylene Packaging Material Using Molecular Dynamics Simulations

**DOI:** 10.3390/polym14091779

**Published:** 2022-04-27

**Authors:** Chandra Mouli R. Madhuranthakam, Sudharsan Pandiyan, Alexander Penlidis

**Affiliations:** 1 Chemical Engineering Department, Abu Dhabi University, Abu Dhabi P.O. Box. 59911, United Arab Emirates; 2 Schrödinger, Bengaluru 560086, India; sudharsan.pandiyan@schrodinger.com; 3 Chemical Engineering Department, Institute for Polymer Research (IPR), University of Waterloo, Waterloo, ON N2L 3G1, Canada; penlidis@uwaterloo.ca

**Keywords:** nonacosan-10-ol, amorphous polyethylene, oxygen diffusion, molecular simulations, glass transition temperature

## Abstract

Packaging material has a significant role in maintaining or altering the shelf life of different products. Polymer materials are extensively used as packaging materials for different perishable and non-perishable products both during transportation and storage. This article aims at developing a new polymer composite which can be used as packaging material. This new composite addresses the challenge of controlling oxygen diffusion rates during the storage of perishable goods such as vegetables, meat and produce, etc. The proposed new composite primarily consists of nonacosan-10-ol and polyethylene. Molecular dynamics simulations (MDS) are performed by mixing 5.2%, 17.1%, 29.2%, 40.8% and 45.2% (wt/wt) of nonacosan-10-ol to amorphous polyethylene. Mechanical properties such as Young’s modulus/glass transition temperature, and gas transport properties such as diffusion coefficient and diffusion volume are estimated from the MDS and diffusion related simulations consisting of different oxygen concentrations in polyethylene-alone system and polyethylene- nonacosan-10-ol blends. The impact of adding different weight percent of nonacosan-10-ol to polyethylene is quantitatively assessed and optimal composition of the proposed additive is suggested corresponding to minimal oxygen diffusion rate, high elastic modulus and good thermal stability.

## 1. Introduction

Polyethylene (PE) films are extensively used as packaging material for both commercial and food products. When packaging materials are used for food products, some of the important properties of interest are mechanical strength, thermal stability, diffusion characteristics of certain compounds such as oxygen, carbon dioxide and ethylene. These properties strongly affect the storage and shelf life of the product being packaged. Extensive research is being conducted to improve the shelf life and minimize the food wastage by making modifications to the packaging material in the form of finding appropriate additives. These additives can either be synthetic chemicals or bio-based chemicals extracted from a natural source. Consumers demand cleaner labels with minimum synthetic chemicals being used as additives in the materials used for packaging food products. This in turn drives the necessity to use natural compounds derived from oils and plant extracts [1,2,3,4]. Ordon et al. [5] found that low density polyethylene films covered with coatings containing extracts of rosemary, raspberry and pomegranate were active against selected bacterial strains and able to extend the shelf life of food products. Ediyilyam et al. [6] used biopolymers such as chitosan and gelatin along with silver nanoparticles in thin film composites targeted to the synthesis of cheap, environmentally friendly, non-toxic packing material. Popovic et al. [7] showed that films made of pumpkin seed oil cake and PE could have good water vapor barrier properties and also oxygen barrier properties along with satisfactory mechanical properties. In this article, it is proposed to use nonacosan-10-ol (interchangeably referred to as ‘wax’) as an additive to polyethylene. Nonacosan-10-ol is one of the kinds of wax derived from plants mainly found in the leaves of Nelumbo nucifera and Pinus halepensis. In Nelumbo nucifera, nonacosan-10-ol is found in the underside of the leaves [8] or in the needles of pine Pinus halepensis [9]. Fernandes and Madhuranthakam [10] found that nonacosan-10-ol-based cellulose composite exhibits excellent mechanical properties and can be used as a sustainable packaging material. To minimize plastic waste and its deleterious impact on the environment there is a continuous demand for developing sustainable packaging material especially for bio-based polymers and/or bioplastics in addition to recycling and reducing the use of virgin polymer materials. 

In this article we report on the mechanical properties and diffusion of oxygen through composite made of polyethylene and nonacosan-10-ol wax. Different weight percent of the nonacosan-10-ol wax is mixed with a specific number of polyethylene chains and molecular dynamics (MD) simulations are performed to assess the performance and stability of this composite. MD simulations is an effective tool for not only predicting the behavior of molecules at small scale but also very useful in estimating mechanical properties of polymeric materials. Since nonacosan-10-ol is mainly composed of carbon atoms, we expect that it can easily fill up the space within the polyethylene matrix and would lead to enhanced mechanical behavior of the material and also affect the barrier properties in terms of lowering the diffusion ability of oxygen molecules. 

## 2. Simulations Details

All molecular dynamics simulations were performed using the Material Science (MS) Suite version 4.4.135 of Schrödinger 2021-4 release (Schrödinger, LLC, New York, NY, USA) which used the OPLS4 force field [11]. Chemical structures of the amorphous polyethylene chains, nonacosan-10-ol and oxygen molecules were drawn using the 2D sketcher which in turn were converted to 3D using the MS Maestro interface. Composites of different weight percent of nonacosan-10-ol in polyethylene were built using the Disordered System builder in the MS Suite. A unit cell with 5 nm × 5 nm × 5 nm was used. Ten linear polyethylene chains each consisting of 662 atoms (112 repeating units and two end H atoms) were used as a base case for the polymer system alone for which 5, 15, 30, 40 and 60 molecules of nonacosan-10-ol were added which led to corresponding wax compositions (on weight basis) of 5.2%, 17.1%, 29.2%, 40.8% and 45.2%, respectively. These structures were initially relaxed and equilibrated for 200 ns using the Multi-Stage Simulation workflow in MS suite. Following are the different steps incorporated during the equilibration of all systems.

- Brownian dynamics for 0.1 ns with NVT ensemble at 10 K.
- MD simulation with a time step of 1 fs with NVT ensemble at 300 K.
- MD simulation with a time step of 1 fs with NVT ensemble at 700 K.
- MD simulation with a time step of 2 fs with NPT ensemble at 300 K and 1.01325 bar.
- MD simulation for 10 ns with NPT ensemble at 300 K and 1013.25 bar.
- MD simulation for 10 ns with NPT ensemble at 300 K and 1.01325 bar.
- Brownian minimization for 100 ps.
- MD simulation for 200 ns with NPT ensemble at 300 K and 1.01325 bar.
- Analysis of bulk properties for the final system.

The stress-strain calculations were performed using the option of volume being conserved in all three directions, with a Poisson ratio of 0.5, a strain rate of 0.02 × 10^8^ s^−1^ and using a strain step size of 0.0015 for 100 steps. These simulations were run for a maximum strain of 0.15 with the objective of estimating the Young’ modulus assuming that the stress-strain curve is linear within this strain limit. The simulation protocol involved using a simulation time of 1000 ps with a time step of 2.0 fs and trajectory recording interval of 10 ps at a temperature of 300 K. For the thermophysical properties, the system is cooled from 200 K to 60 K in steps of 5 K constrained to convergence from each of the previous temperature steps. The simulation time was 10 ns at a pressure of 1.01325 bar, for 3 maximum cycles and 5% convergence. Both stress-strain and thermophysical properties would lead to an understanding of the performance and stability of the PE-wax blends, which is important when they are used as packaging materials. In addition to the mechanical properties, diffusion of oxygen through the PE-wax blend was also studied. For the simulations related to oxygen diffusion through the PE-nonacosan-10-ol composite, diffusion coefficient calculation based on the mean square displacement was used with a linear fit corresponding to simulation time difference (tau) values of 0.01 to 0.99 ns. The diffusion simulations were repeated three times with different initial random velocities of the atoms in the system and the average value of the diffusion coefficient was estimated. 

## 3. Results and Discussion

**Figure 1:**
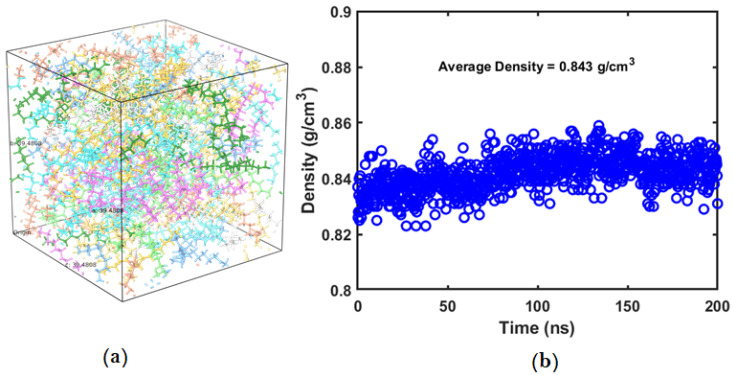
(**a**) Equilibrated polyethylene system with 10 chains (each chain represented with a specific color). (**b**) Density vs. equilibration time profile for polyethylene system.

PE chains used in the simulations were longer compared to the wax molecule, and hence all systems were equilibrated for at least 200 ns, and the resultant equilibrated systems were used for estimating other mechanical properties. The first attribute used to a confirm relaxed equilibrated system is the steady profile obtained for density/volume of the system. The density of the amorphous PE system depends on the initial number of united atoms present, number and type of chains. After equilibrating, the density of the amorphous PE system was found to be 0.843 g/cm^3^, which is very close to the value reported in the literature [12]. Figure 1a shows the relaxed and equilibrated amorphous PE system, and Figure 1b shows the corresponding density variation with respect to the equilibration time. The density of crystalline PE will be approximately 0.94 gm/cm^3^, which is greater than the density of amorphous PE.

**Figure 2:**
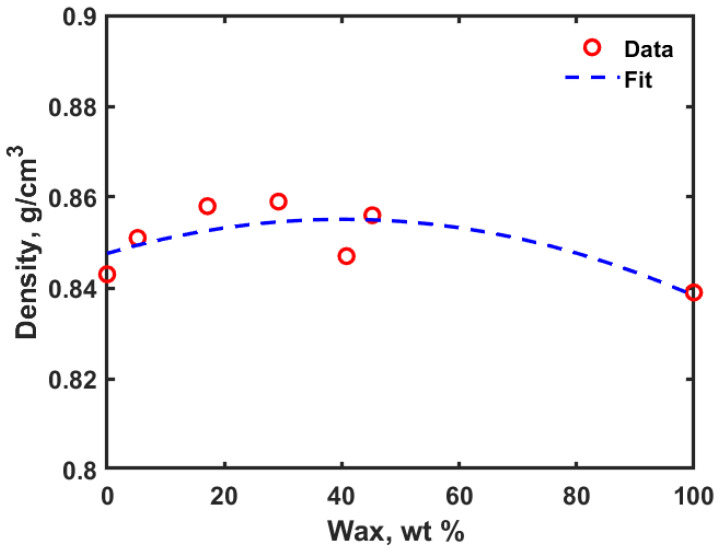
Density versus weight percent of wax in the PE-nonacosan-10-ol systems.

MD simulations of the PE embedded with different compositions of nonacosan-10-ol showed that the density slightly increased initially and then decreased with an increase in the weight percent of the wax (as shown in Figure 2). 

**Figure 3:**
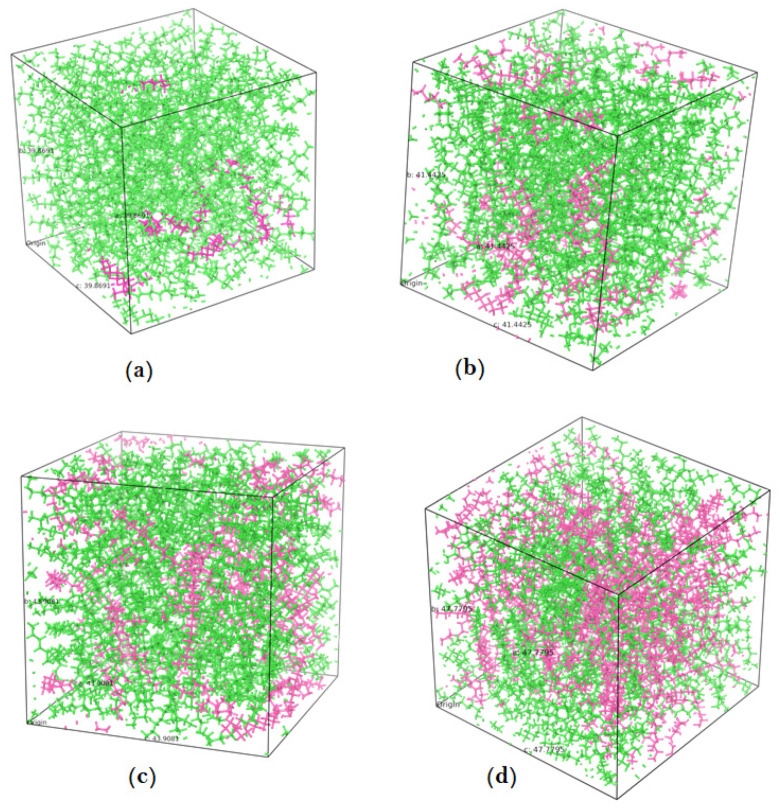
Equilibrated PE-wax system (**a**) 5.1 wt% wax (**b**) 17.1 wt% wax (**c**) 29.2 wt% wax and (**d**) 45.2 wt% wax (PE chains are green, wax molecules are pink).

Density of the wax molecules was obtained to be 0.839 g/cm^3^, which is very close to the literature reported value of 0.84 g/cm^3^ [13]. The density of pure wax is less than the density of the amorphous PE. It was observed that the initial free volume which is available in the PE matrix was occupied by the nonacosan-10-ol molecules. As the concentration of the nonacosan-10-ol was increased from 5.2% to 45.2%, the available free volume within the PE matrix decreased. At higher weight percentages of wax, the contribution towards the density of the composite is more pronounced compared to that of lower percentages of wax. Figure 3a through Figure 3d shows the equilibrated PE-wax system for different compositions of nonacosan-10-ol.

### 3.1. Stress-Strain Results

**Figure 4:**
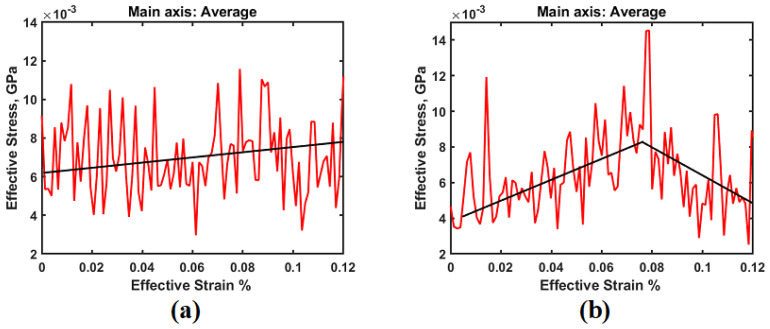
(**a**) Stress vs. strain for PE alone 3. (**b**) Stress vs. strain for PE-wax (45.2 wt%).

**Figure 5:**
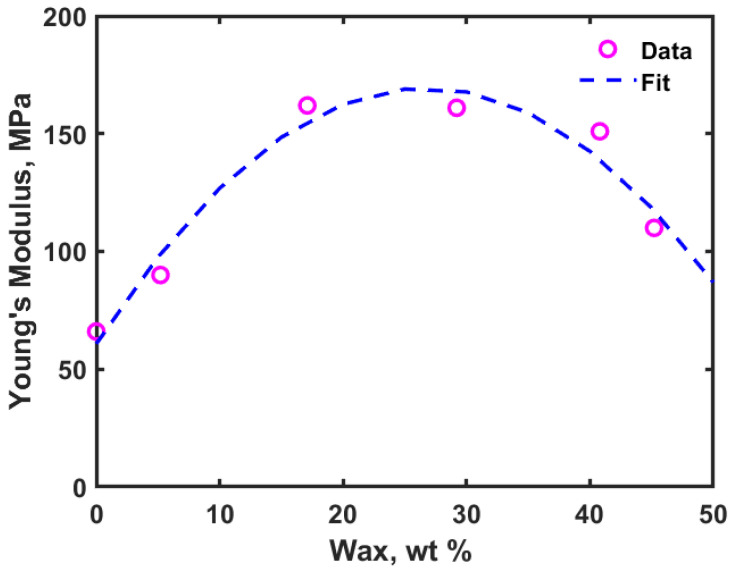
Young’s modulus versus weight percent of PE-wax blends.

Nonacosane-10-ol is a plant-based wax and offers least mechanical strength when it is used alone for any application. When we add this wax to PE, mechanical properties are expected to be affected [10]. Djokovic et al. [14] conducted experiments with low density polyethylene (LDPE) and oxidized Fisher-Tropsch wax blends. They reported that the blends showed improved mechanical properties if a small amount of wax (up to 10 wt%) was added to PE. In this article, we use a natural wax obtained from natural plant sources, as mentioned in the introduction section. Figure 4a shows the stress-strain curve for PE alone, while Figure 4b shows the stress-strain curve obtained for PE-45.2 wt% wax blend (similar plots have been obtained for other PE-wax blends). In the elastic regime (also called regime I), where the stress increases linearly with strain, the corresponding slope of the linear curve was used to calculate the Young’s modulus. Figure 5 shows the results obtained for the Young’s modulus for all different blends considered in this study.

From the stress-strain results, one can see that adding wax to PE has significantly affected the mechanical properties of the blend. The Young’s modulus was generally observed to be greater for the PE-wax blends compared to PE alone. From Figure 5, upon increasing the wax content, the Young’s modulus increases up to 29.2 wt% wax content, and subsequently decreases with further addition of wax to PE, although still greater than that of PE alone. Djokovic et al. [14] also observed the same trend but in their case the Young’s modulus was continuously increasing with increase in the percentage of oxidized Fisher-Tropsch wax added to the low density polyethylene. The most likely reason for the observed trend is that with an increase in the wax content, the system tends to form or approach a more crystalline state (compared to the incipient purely amorphous state). Further, at higher concentrations of wax, the amorphous regions within the matrix are affected as wax will try to increase the ability to flow due to a decrease in the overall viscosity. Based on the values obtained for Young’s modulus, it was observed that adding up to 29.2 wt% nonacosan-10-ol to the PE would lead to higher modulus of elasticity for the blends compared to the PE alone scenario.

### 3.2. Glass Transition Temperature (T_g_) Results

**Figure 6:**
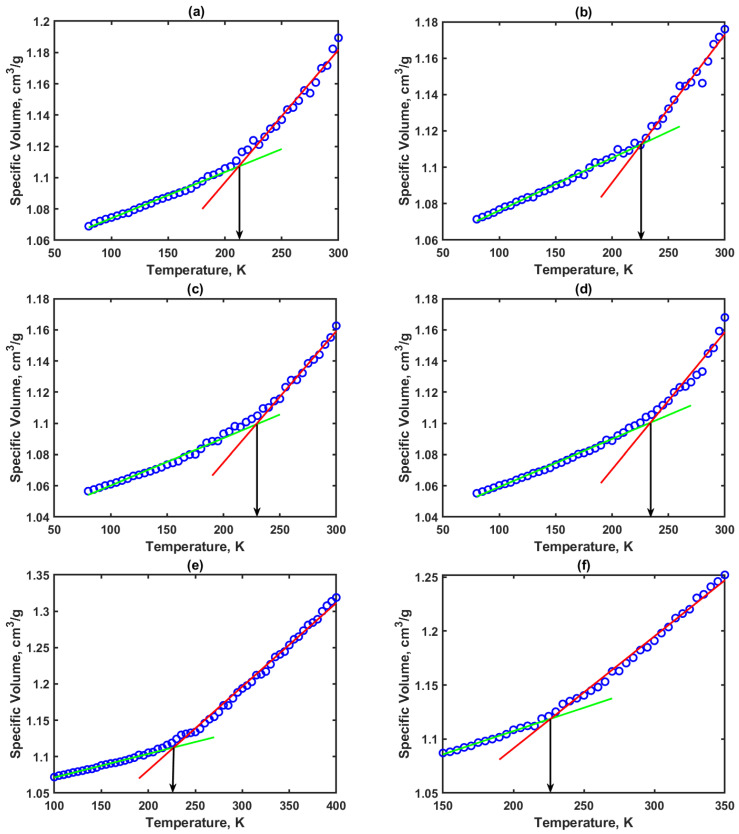
Specific volume vs. temperature profiles for evaluation of T_g_: (**a**) PE alone; (**b**) PE and 5.2 wt% wax; (**c**) PE and 17.1 wt% wax; (**d**) PE and 29.2 wt% wax; (**e**) PE and 40.8 wt% wax; (**f**) PE and 45.2 wt% wax (green straight line represents glassy region fit and red straight line represents rubbery region fit).

**Figure 7:**
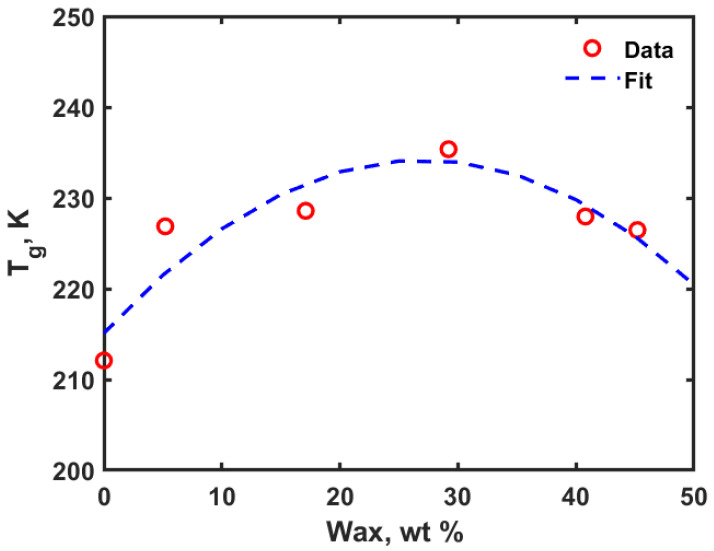
Glass transition temperature versus weight percent of wax in polymer blends.

From the thermophysical property simulations, the relationship between the specific volume of the system (PE or PE-wax blend) and temperature was obtained. A bilinear piecewise fitting was used to capture the pattern obtained in these simulations with the main objective of estimating the glass transition temperature which indirectly tells about the thermal stability of the PE-wax blends. Figure 6a through Figure 6f shows the specific volume vs. temperature profiles obtained for the PE and PE-wax blends and Figure 7 shows the T_g_ versus weight percent of the wax. 

For the amorphous PE alone system, T_g_ was obtained to be 212 K, which is very well within the range of values reported in the literature. For amorphous PE, there has been a wide range of T_g_ values (−35 °C to −135 °C) reported in the literature [15,16]. This is due to the fact that PE with linear (unbranched), almost perfect chain structure, has high crystallization rates, and this makes it very hard to capture transitions in its amorphous state. Yet, the T_g_ value of 212 K obtained for PE alone considered in this study with 10 chains of 200 monomer units is very close to the MDS value for the amorphous PE (with two chains of 300 repeat units) reported by Yang et al. [17], which was 200 K. As shown in Figure 7, with an increase in the wax content the T_g_ increased compared to the amorphous PE case up to wax addition of 29.2 wt% and then T_g_ started to decrease with further increase in the wax content in the PE matrix. This shows that PE with wax content up to 45.2 wt% led to an increase in the T_g_ within a range of 10 to 20 K. Hence, the PE-wax blends are thermally stable and can be conveniently used as packaging material for applications that require temperatures as low as 226 K. 

### 3.3. Diffusion Coefficient Results

**Figure 8:**
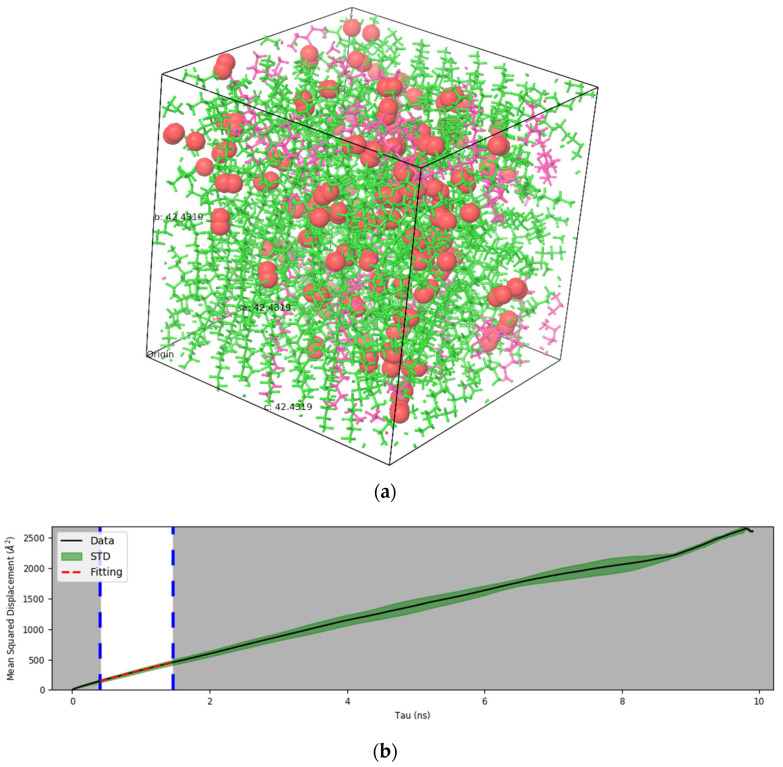
(**a**) PE-17.1 wt% wax and oxygen (red spheres represent oxygen atoms). (**b**) Mean square displacement curve corresponding to the PE-17.1 wt% wax and oxygen.

Oxygen diffusion through the PE-wax blends is an important characteristic that will affect performance when used as packaging materials, especially for storing perishable goods. Other components such as carbon dioxide, nitrogen and ethylene may also affect barrier properties. In this article, we report the diffusion of oxygen through different PE-wax blends. Initially, the self-diffusion of oxygen was studied by considering different numbers of oxygen molecules within a given volume and it was found that 5 vol% of oxygen which is equivalent to 100 molecules would behave as a bulk system. Hence, the same number of oxygen molecules were used to perform MD simulations for PE alone, PE-wax blends and nonacosane-10-ol alone. The corresponding oxygen diffusion coefficient is calculated using the Einstein method, which uses the mean square displacement (MSD) curve obtained for different molecules of the system. For all systems, the MSD curve has a linear part and a nonlinear part. The nonlinear part happens at longer times and is due to unstable vibrations and noise. Using the linear part of the MSD, on can calculate the corresponding diffusion coefficient. All diffusion simulations were repeated for a minimum of three times and the average value for the diffusion coefficient of oxygen (D) was calculated. Figure 8a shows the PE-wax with oxygen for 17.1 wt% wax (as a sample case), whereas Figure 8b shows the corresponding MSD obtained for this system from which D was estimated.

Table 1 shows the summary of the diffusion coefficients obtained for the different PE blends, including the cases of PE alone and wax alone systems.

**Figure 9:**
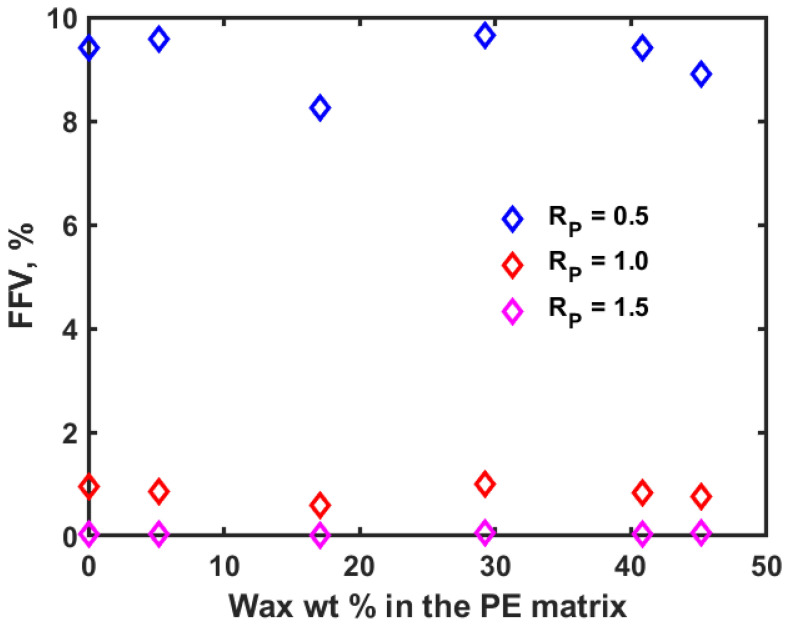
Fraction of free volume in % vs. the weight % of wax in the PE matrix with different probe radii (R_P_ in Å).

The diffusion coefficient of oxygen at 300 K and 1.01325 bar in amorphous PE obtained in the current study is very close to the reported value of 5.689 × 10^−10^ m^2^/s [18]. The diffusivity of oxygen in the PE-wax matrix decreased with an increase in the wax content of the PE matrix as shown in Table 1. The decrease in diffusivity is low at lower wax content but increases as the wax content reaches 29.2%. In the effort of using these blends as packaging material, in addition to having good mechanical and thermal stability, they would also offer the benefit of decreasing the diffusion of oxygen, which would enable an increase in the shelf life of some of the stored perishable products. The variation in the diffusion coefficient in the polymer matrix with wax can be understood further by estimating the fraction of free volume (FFV). FFV is defined as the ratio of the free volume to the simulated volume where the simulated volume is the sum of the free volume and volume occupied by the material. With MD simulations, in addition to estimating the FFV, the shape and size of the free volume can also be visualized and analyzed. The percentage of free volume can be obtained by using a molecular probe with certain radius (R_P_) that moves over the van der Waals surface of the matrix within the unit cell. In this study, different R_P_ values (0.01 Å, 0.5 Å, 1.0 Å, 1.5 Å, and 1.7 Å) are used to confirm the observed variations in the diffusion coefficient estimations. Figure 9 shows the estimated FFV using different R_P_ values of 0.5 Å, 1.0 Å and 1.5 Å for the PE-wax blend system. Since the atomic radius of carbon and oxygen are 170 and 152 pm, respectively, a molecular probe with R_p_ value less than or equal to 1.52 Å would be apt for finding the FFV in the context of studying the diffusion of oxygen through the PE-wax matrix. From Figure 9, it is evident that as the probe radius increases, the FFV decreases, and the corresponding diffusion coefficient decreases accordingly (see again Table 1).

Finally, from Figure 9, when wax is added to the PE system, the initial free volume present with the PE matrix becomes occupied by the wax molecules due to which the FFV decreases, and this in turn causes a decrease in the diffusion of oxygen. When the wax concentration is increased beyond 29.2%, the free volume available becomes almost constant and the corresponding diffusion of oxygen also reaches an almost steady value. Based on the results obtained, for higher Young’s modulus, moderate T_g_ value and low diffusion coefficient of oxygen, PE with 17.1 wt% wax can be the optimal choice while adding up to 29.2 wt% will also be applicable if higher T_g_ value is allowed in the corresponding application.

## 4. Conclusions

A new packaging material consisting of polyethylene and plant-based wax, nonacosane-10-ol, was designed and studied using molecular dynamics simulations. Different amounts of wax were combined with the PE and the corresponding performance and stability in terms of stress-strain properties, thermophysical characteristics and diffusion of oxygen were considered. It was found that by adding wax, the Young’s modulus of the PE composite increased up to 29.2 wt% of wax, whereby after this the increase in Young’s modulus was moderate, though still higher than that of the PE alone. The glass transition temperature of the wax-based PE composite increased up to 235 K corresponding to 29.2 wt% wax in the PE and was found to be closer to the PE alone case when the wax percentage reached 45 wt%. The diffusion coefficient of oxygen in the PE-wax blends decreased up to 10 to 15% compared to the diffusion coefficient of oxygen in PE system alone. Based on the results obtained from these studies, the proposed new packaging material based on PE and nonacosane-10-ol offers many benefits with respect to better mechanical properties and lower oxygen diffusion rates. This new material can be used for both low temperature and room temperature storage of perishable products and quite efficiently for longer times due to lower oxygen diffusion rates. 

## Figures and Tables

**Table 1:** Diffusion coefficient of oxygen from MD simulations.

**Weight % of PE-** **Nonacosan-10-ol**	**Diffusion Coefficient D, m^2^/s**	**% Decrease in D with** **Respect to PE**
100–0	5.062 × 10^−10^ ± 1.2067 × 10^−11^	-
94.8–5.2	4.7158 × 10^−10^ ± 1.089 × 10^−11^	6.83
82.9–17.1	4.6547 × 10^−10^ ± 1.6711 × 10^−11^	8.05
70.8–29.2	4.2289 × 10^−10^ ± 1.5893 × 10^−11^	16.46
59.2–40.8	4.3856 × 10^−10^ ± 9.5663 × 10^−12^	13.36
54.8–45.2	4.3060 × 10^−10^ ± 7.6644 × 10^−12^	14.94
0–100	1.0159 × 10^−9^ ± 1.7738 × 10^−11^	-
